# Sex Difference in All-Cause and Infection-Specific Mortality Over 10 Years Post Hip Fracture

**DOI:** 10.1093/geroni/igab046.627

**Published:** 2021-12-17

**Authors:** Rashmita Bajracharya, Jack Guralnik, Jay Magaziner, Michelle Shardell, Alan Rathbun, Takashi Yamashita, Denise Orwig

**Affiliations:** 1 University of Maryland, School of Medicine, Catonsville, Maryland, United States; 2 University of Maryland, Baltimore, Baltimore, Maryland, United States; 3 University of Maryland School of Medicine, Baltimore, Maryland, United States; 5 University of Maryland Baltimore, Baltimore, Maryland, United States; 6 University of Maryland, Baltimore County, Baltimore, Maryland, United States

## Abstract

Men die at a twice higher rate than women in the first two years after fracture and also experience higher infection-related mortality. Most research has only looked at differences in short-term mortality after hip fracture. The objective was to determine if cumulative incidence of all-cause mortality and infection-specific mortality is higher in men compared to women over ten years. Data came from Baltimore Hip Studies7th cohort. Women were frequency-matched (1:1) to men on timing of fracture to ensure equal numbers of men and women. The association of sex and all-cause mortality was analyzed using Cox proportional hazard model and a cause-specific hazard model for infection-specific mortality. Both models controlled for age, cognition, comorbidity, depressive symptoms, BMI, and pre-fracture ADL limitations. Complete-case sample size was 300 (men=145, women=155). By the end of ten years from the date of admission for a hip fracture, there were 237 (men=132, women=105) all-cause deaths and 38 (men=25, women=13) infection-specific deaths. Men had significantly higher all-cause mortality risk [73.7% vs 59.3%; HR=2.31(2.02-2.59)] and infection-specific mortality [17.2% vs 8.3%; HR=4.43(2.07-9.51)] compared to women. In addition to sex, older age, cognition, and comorbidities were associated with all-cause mortality whereas only BMI was associated with infection-specific mortality in adjusted models. Men had a higher risk of mortality over 10 years compared to women, specifically two-fold higher risk of infection-specific mortality compared to all-cause mortality. Findings imply that interventions to prevent/treat infection, tailored by sex, may be needed to narrow significant differences in long-term mortality rates between men and women.

